# Social Health and COVID-19 Pandemic

**DOI:** 10.18502/ijph.v49i10.4715

**Published:** 2020-10

**Authors:** Dariush D. FARHUD

**Affiliations:** 1. School of Public Health, Tehran University of Medical Sciences, Tehran, Iran; 2. Departments of Basic Sciences/Ethics, Iranian Academy of Medical Sciences, Tehran, Iran; 3. Farhud Genetic Clinic, Tehran, Iran

With the incidence and prevalence of COVID-19 disease, health, social, and economic considerations need to be reviewed and redefined. Since this pandemic, even more severe and pervasive than the First and Second World Wars, has disrupted the world’s social systems and economic structures, many well-known concepts and disciplines in the social health sciences need to be adapted. Changes in many professions, industries and economic resources have undoubtedly made new demands on social health.

Adherence to the principles of individual ethics, professional ethics, and social ethics, attention to social media (in economics, education, etc.), sustainable development, social justice, prevention of violence (family, social, governmental), vigilance and active management of international institutions (United Nations, UNESCO, World Health Organization, etc.) in preventing wars between governments, equitable distribution of food resources, preventing the increase of non-communicable diseases (cardiovascular disease, cancers, metabolic diseases, mental disorders) are among the urgencies of government and global management.

Here it is necessary to mention a group of disciplines discussed in social health (Table 1).

**Table 1:** Different groups of disciplines discussed in social health

***Discipline***	
Addiction	Health Education
Aging and Society	Health Habits/ Health Illiteracy
Anthropology	Health Policy and Practices
Behaviorology	Human Ecology
Circadian Rhythm / Biological Clock	Justice in Social Health
Clinical Psychology	Life Style, Life Skills, Quality of Life
Cognitive sciences	Medical Sociology
Communicable Diseases (Epidemic and Pandemic)	Mental Health
Communication	Non-communicable Diseases (NCDS)
Developmental Psychology	Occupational Health
Emotional Support	Peace, Freedom
Environmental Emergency	Personalized/precision medicine
Environmental Health	Physical Health
Epigenetics	Public Health
Ethical Issues	Social Empathy
Family Life (Resilience, Adaptability, Loneliness, Depression)	Social Isolation
Food and Nutrition Security	Social Psychology
Food Habits	Social Relationships
Gender and Racial Equality	Social Support
Genetic Disorders	Social Ties
Health Behavior	Social Welfare / Social Well-Being
Health Care	Sustainable Development
Health Economy	

